# Estimating Effective Population Size from Linkage Disequilibrium between Unlinked Loci: Theory and Application to Fruit Fly Outbreak Populations

**DOI:** 10.1371/journal.pone.0069078

**Published:** 2013-07-23

**Authors:** John A Sved, Emilie C. Cameron, A. Stuart Gilchrist

**Affiliations:** 1 School of Biological, Earth and Environmental Sciences, University of New South Wales, Sydney, New South Wales, Australia; 2 Faculty of Health, University of Newcastle, Callaghan, New South Wales, Australia; National Institute of Environmental Health Sciences, United States of America

## Abstract

There is a substantial literature on the use of linkage disequilibrium (LD) to estimate effective population size using unlinked loci. The 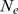 estimates are extremely sensitive to the sampling process, and there is currently no theory to cope with the possible biases. We derive formulae for the analysis of idealised populations mating at random with multi-allelic (microsatellite) loci. The ‘Burrows composite index’ is introduced in a novel way with a ‘composite haplotype table’. We show that in a sample of diploid size 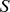, the mean value of 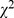 or 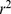 from the composite haplotype table is biased by a factor of 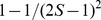, rather than the usual factor 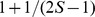 for a conventional haplotype table. But analysis of population data using these formulae leads to 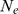 estimates that are unrealistically low. We provide theory and simulation to show that this bias towards low 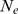 estimates is due to null alleles, and introduce a randomised permutation correction to compensate for the bias. We also consider the effect of introducing a within-locus disequilibrium factor to 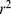, and find that this factor leads to a bias in the 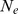 estimate. However this bias can be overcome using the same randomised permutation correction, to yield an altered 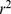 with lower variance than the original 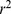, and one that is also insensitive to null alleles. The resulting formulae are used to provide 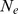 estimates on 40 samples of the Queensland fruit fly, *Bactrocera tryoni,* from populations with widely divergent 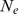 expectations. Linkage relationships are known for most of the microsatellite loci in this species. We find that there is little difference in the estimated 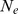 values from using known unlinked loci as compared to using all loci, which is important for conservation studies where linkage relationships are unknown.

## Introduction

The magnitude of linkage disequilibrium (LD) can be used to estimate effective population size [1]–[5]. In general, low populations sizes are expected to give rise to relatively high levels of LD, and similarly high population sizes to low LD levels. An important feature of this means of estimation is that measurement at a single point in time can provide information on effective size. Furthermore closely-linked loci give information on population sizes over historical periods of time, while loosely-linked loci estimate population sizes in the immediate past [3], [4].

Much recent attention has been paid to the use of unlinked loci for estimating population size, for which the term ‘Linkage Disequilibrium’ will inappropriately be used. There are three major advantages of studying unlinked loci. First, the majority of pairs of loci are unlinked. Secondly, these are the only locus pairs for which it is easy to estimate the recombination frequency, 50%. Finally, in the study of pest populations, and in the area of conservation, it is usually the most recent population sizes that are of interest, for which unlinked loci are the most relevant.

The principal problem in studying unlinked loci comes from the sample sizes needed to obtain accurate LD estimates. The expected disequilibrium is a function of 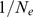, where 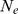 is the effective population size, assumed constant, and 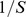, where 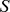 is the sample size [6]. Unless sample sizes are large, the latter can overwhelm the former.

A second complication comes from the usual necessity to use diploid data. Most LD theory is based on haplotypes rather than diploid genotypes, which typically cannot be observed. Although the recognition of haplotypes may seem inappropriate for unlinked loci, the same distinction applies as for linked loci, because the information on population size comes from genes with the same parental origin rather than genes inherited from different parents. The passage from zygotic to to gametic parameters can be made using either the maximum likelihood estimator of Hill [7], or, as will be used here, the Burrows estimator as elaborated by Weir [8].

In preliminary investigations of the size of Queensland fruit fly populations, we found very low 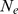 estimates for populations that are believed to be large. We traced this discrepancy to an excess of homozygous genotypes, believed to be due to the presence of null alleles at some of the microsatellite loci used in the study.

Because of these complications, the problem of finding an adequate estimator of 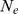 is fraught with potential biases. Waples and Do [9] have, however, shown that their LDNe program works well in estimating 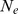 from simulated data. The program uses empirically derived correction factors rather than investigating the underlying reasons for the biases. The purpose of the present paper is to produce an analytical solution to account for the biases. We derive two sets of formulae that do this, depending on whether a ‘within-locus disequilibrium factor’ is used or not, and compare the application of these two sets to simulated and real data.

## Materials and Methods

### Queensland Fruit Fly Samples

Two data sets are analysed in the paper.

1. East coast Australian populations. The data are from 55 samples from towns in the state of NSW in the years 2002–2004 [10]. Some of these sample come from areas where the flies are endemic, and in other cases the outbreaks appear to be only temporary.
2. NorthWest. These flies were collected during the years 1999–2003 from Northern West Australia and the Northern Territory [11].

The data in the two cited papers have previously been summarised only in terms of single locus statistics. The present paper involves a two-locus analysis, which requires additional information from the original data sets. The original data sets are provided in Supporting Information, Data S1 and Data S2.

### Computer Simulation

All simulations reported in the paper are forward Monte-Carlo simulations under the Wright-Fisher model. Parents were chosen randomly in each case, thereby allowing selfing and not assuming permanent mate bonding, an important aspect of population structure [6]. Most simulations involved a starting population with either 16 or 32 loci, each locus having the number of alleles chosen randomly between 2 and 8. Alleles were assigned randomly at different loci, assuming no systematic LD. Populations were simulated for 20 generations, followed by sampling without replacement of 32 individuals from the final population, and calculation of LD levels. Simulations were written in C, and are available on request.

### Theory

Most of LD theory applies to gametes rather than genotypes. Fortunately a simple method, the ‘Burrows composite LD coefficient’, is available for handling genotypes. This coefficient has been defined by Cockerham and Weir [12] in terms of sums of genotype frequencies. It is convenient to introduce here a slightly different but simpler way of relating genotype frequencies to gamete frequencies. See Table 1 for a listing of symbols used.

**Table 1:** Symbols used in the text.

N_e_	Effective population size
S	Number of diploid individuals in a sample
n_11_	Number of genotypes in a sample with aa at first locus and bb at second locus
n_12_	Number of aa b– genotypes where – refers to non-b allele at the second locus
n_21_	Number of a– bb genotypes
n_22_	Number of a– b– genotypes
n_a_, n_b_	Number of a and b alleles respectively
p_a_, p_b_	Allele frequencies in gametic and composite table, = n_a_/2S and n_b_/2S
p_ab_	Frequency of the ab haplotype
D	Gametic disequilibrium coefficient = p_ab_ – p_a_p_b_
r^2^	Gametic correlation = D^2^/[p_a_(1– p_a_)p_b_(1– p_b_)]
M	Number of ab haplotypes in composite haplotype table = 4n_11_+2n_12_+2n_21_+ n_22_
p_ab_(comp)	Frequency of ab in composite haplotype table = M/4S
D(comp)	Disequilibrium coefficient from composite haplotype table = p_ab_(comp) – p_a_p_b_
Δ	Burrows’ disequilibrium coefficient = 2D(comp)
r^2^(comp)	r^2^ value from composite haplotype table = D^2^(comp)/[p_a_(1– p_a_)p_b_(1– p_b_)]
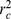	Composite r^2^ parameter = 4r^2^(comp)
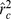	Estimate of 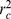 from sample
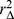	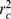 with single-locus disequilibrium = 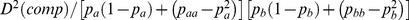
?^2^(comp)	?^2^ calculated from composite haplotype table
p_n_	Frequency of null alleles at a locus
α	Half the difference between coupling and repulsion heterozygote frequencies

**Figure 1:**
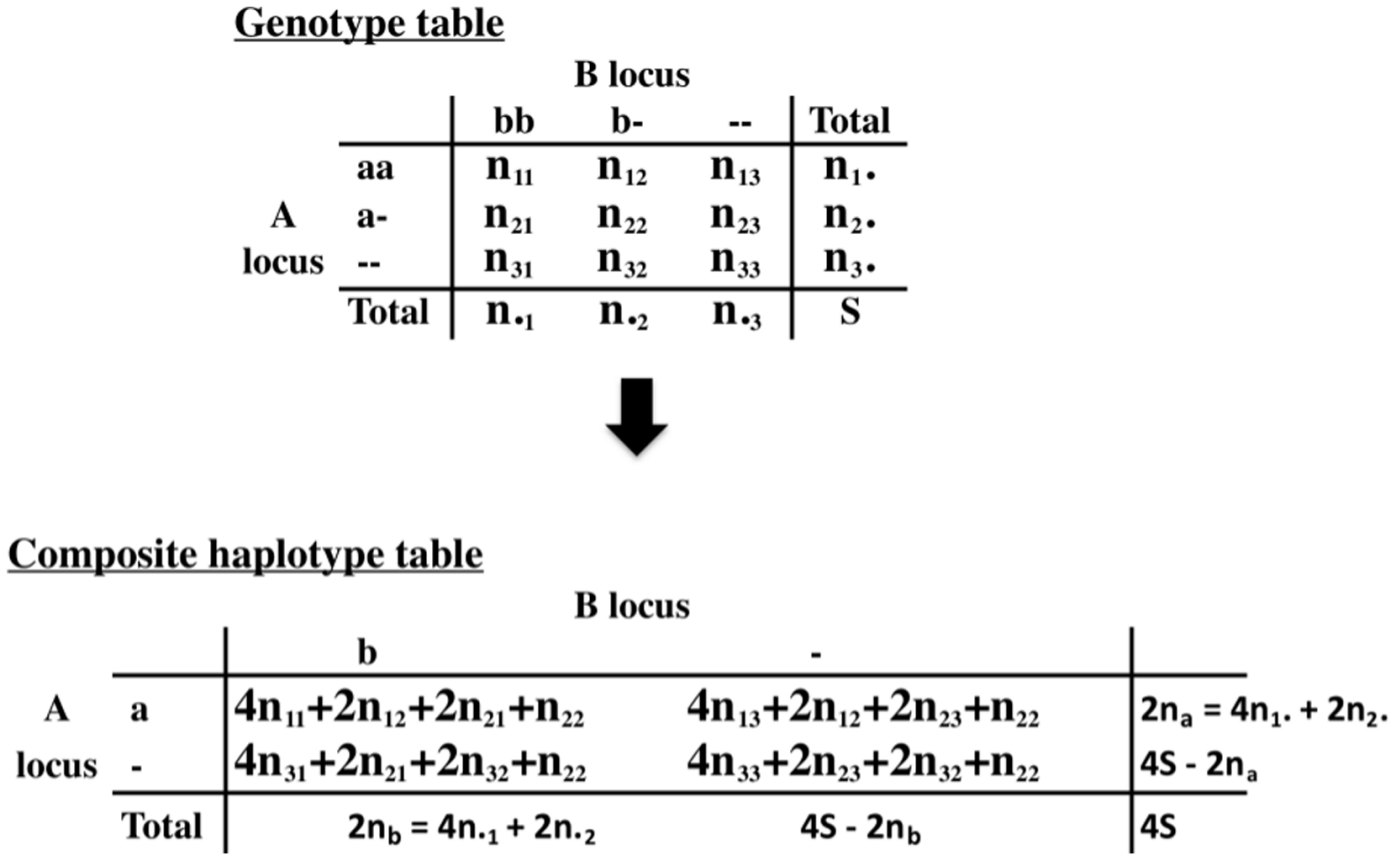
The composite haplotype table for a 2-allele observed sample.

Figure 1 shows the principle for populating a ‘composite haplotype table’. Each genotype in Part (i) contributes the four possible gametes to the composite haplotype table in Part (ii). In the case of double heterozygotes, where the phase is usually unknown, each of the four possible haplotypes is represented. For all other genotypes the haplotypes are known, but each genotype nevertheless contributes four haplotypes. Note the use of 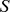 rather than 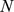 for the diploid sample total to emphasise the distinction between number in a population (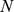) and number in a sample (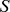). The normal haploid table cannot be written down from the genotypes in Figure 1, but the total would be 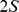, and, for example, the number of 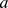 genes = 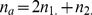. The marginal totals in the composite table are double these.

**Figure 2:**
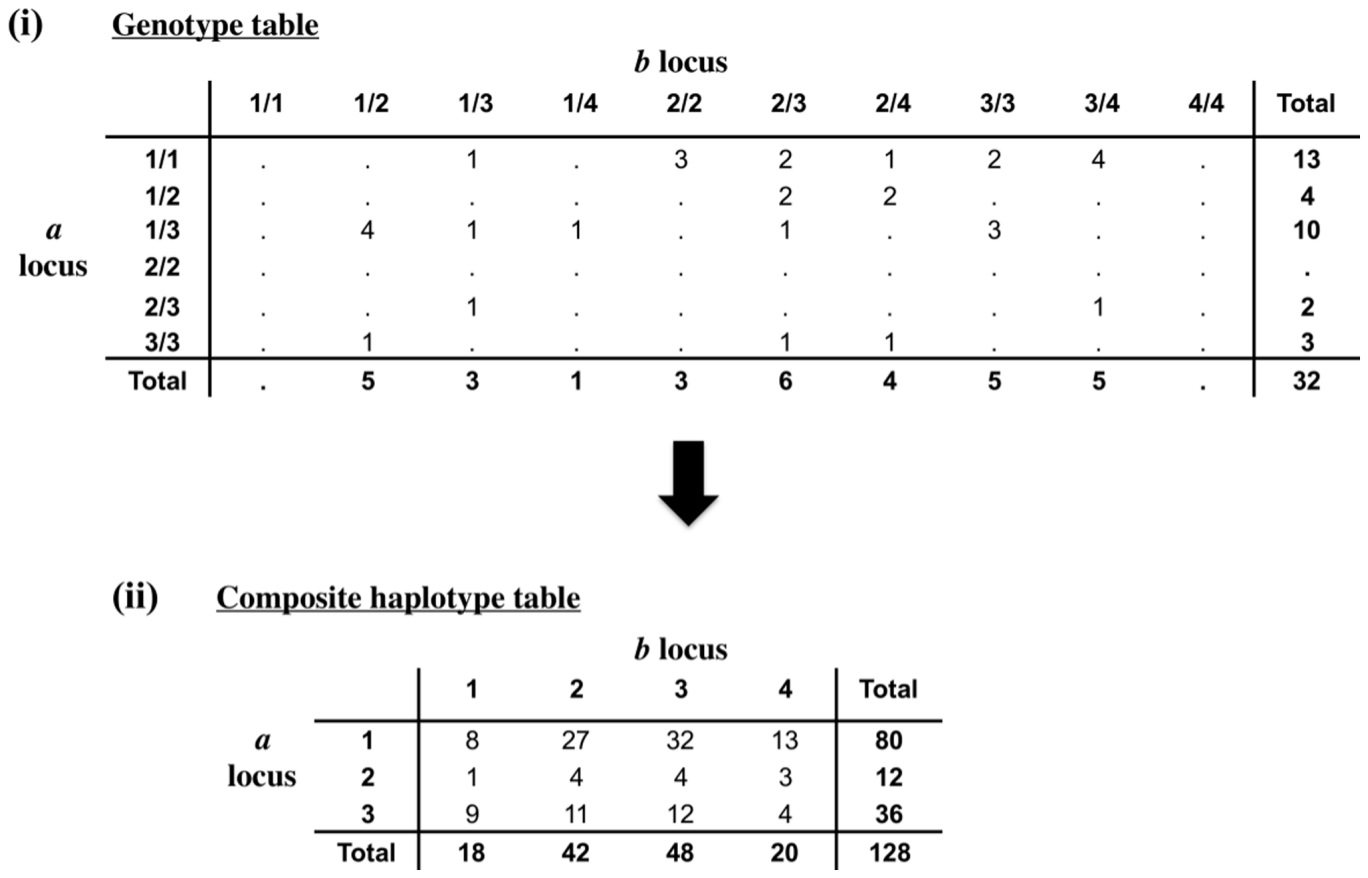
The composite haplotype table for an example of two microsatellites from the fruit y outbreak data set.

Figure 2 shows a numerical example of the composite haplotype table for one sample of size 32 from the Eastern Australia fruit fly data set, where one microsatellite, 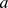, has 3 alleles and a second, 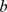, has 4. Again the total in the haplotype table of Part (ii) of Figure 2 is 4x the total in the genotype table of Part (i), rather than 2x as would be found in a table where all haplotypes were known.

The usual LD coefficient can be calculated for the numbers in the composite haplotype table of Figure 1, and given the designation 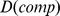. It is:
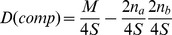



The LD coefficient of Cockerham and Weir [12], 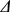, is defined in terms of frequencies 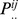 and 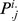, and given as the sum of two coefficients, 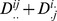:
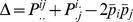



It can be seen from the definitions of 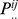 and 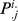 from [12], ignoring the sample-size correction N/(N−1), that this LD coefficient is double the value of 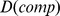 given above.

The intuitive justification for the composite haplotype table is most readily seen in the case of random mating (which is not assumed in the definition of 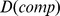). In a genotype such as 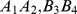, the true haplotypes will be either 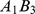 and 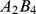 or alternatively 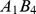 and 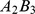. Under random mating, whichever are the ‘false’ haplotypes are expected to occur at frequencies that are simply the products of the relevant gene frequencies. The frequencies contributed by the false haplotypes will dilute, but not bias, the haplotype frequencies. It is readily shown that this dilution will be simply a factor of 2. For example, following Figure 1, the frequency of the 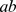 haplotype in the composite table, 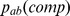, is the true frequency of the 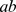 haplotype, 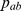, except for the contribution from the double heterozygotes. The true contribution ought to be 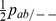, whereas it is in fact 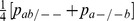. Thus the difference between these two is the difference between 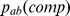 and 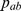, giving.
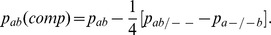



Under the assumption of random mating, it can be seen that.
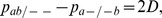
where D is the usual LD parameter, equal to 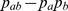. Therefore



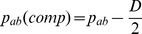



Subtracting 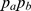 from each side,
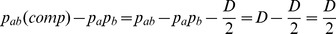



The LHS of this equation is, by definition, the disequilibrium coefficient from the composite table, 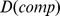. So the equation is simply
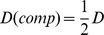



Since this is an expectation under the assumption of random mating, the equation can be written as:
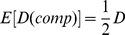
(1)where the expectation is taken over replicate populations of the same sample size.

The LD measure introduced by Hill and Robertson [13] is 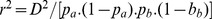. An equivalent parameter can be calculated from the composite haplotype table. The marginal frequencies are the same as for the regular gamete table. So from (1) it follows that the expectation of 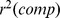 calculated from the composite table is
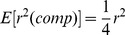
(2)


It is convenient to define a coefficient where, under random mating, the composite 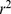 estimates the gametic 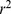, rather than one-quarter of the latter. As pointed out above, the LD coefficient of Cockerham and Weir [12] does this. Therefore we define the statistic 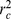 as
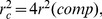
(3)which from (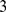), (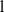) and (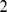) is calculated as



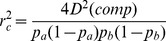
(4)The above definition of 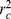 ignores an extra factor introduced by Weir [8]. This factor arises from the potential covariance of the two alleles at the 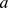 locus and similarly at the 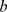 locus. These covariances are implemented through a ‘single-locus disequilibrium factor’, 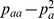 at the 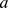 locus and 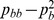 at the 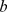 locus, which essentially measure deviations from expected homozygosity. The modified definition of 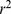, 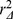, is
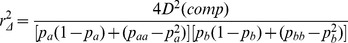
(5)


Because of difficulties in implementing this disequilibrium factor, its discussion is deferred to a later section under this label.

#### 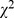 for the composite haplotype table

Owing to double-counting of genes, the composite gamete table has the property that all marginal totals are multiples of 2, while the overall total is a multiple of 4. Nevertheless a regular 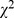 can be calculated for such tables, and the resulting 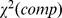 values for a 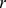
×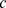 table has close to the expected distribution for 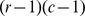 degrees of freedom (Appendix S1). It has the advantage of having more power than the 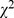 values calculated from the genotype table, owing to the large number of zero and unit values in the genotype table. Its use in independence tests may, however, be limited by its sensitivity to null alleles (see below).

#### Weighting of 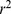 values

The calculation of LD for a microsatellite data set involves two levels of summation. There will usually be many loci, say 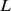, and each of the 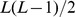 pairs yields a separate estimate of 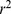. However within each locus pair, say locus 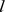 and locus 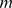, there will be separate calculations for each pair of alleles. These two levels may be labelled as ‘between locus pairs’ and ‘within locus pairs’. Each needs to be separately treated in terms of weighting of the 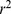 values.

##### Between locus pairs

It is often the case that, through missing readings, different locus pairs will have reduced numbers of observations. The sample size for loci 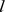 and 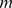 may be designated as 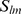. Furthermore some loci will have large numbers of alleles and therefore provide more information than loci with small numbers of alleles. Waples and Do [9] have suggested the weighting 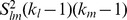 for the different 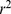 values, where 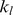 and 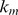 are the number of alleles at the 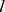 and 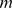 loci respectively. The overall estimate of 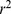 then becomes
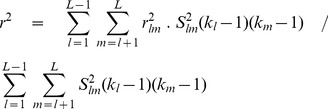
(6)


A recent publication [14] suggests a slightly different weighting compared to that of Waples and Do [9], which would make a small difference to the overall 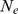 estimate.

##### Within locus pairs


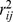
 values for alleles 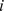 at locus 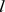 and 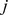 at locus 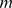 can be simply averaged to provide the 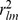 value. However this has the undesirable property that rare alleles exert a disproportionate influence on the overall 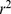 value. This effect that can be ameliorated by omitting low frequency alleles [9]. A more systematic way of avoiding this problem is to weight alleles according to their frequency. In the case where the frequencies of alleles 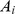 and 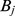 are respectively 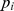 and 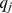, a suitable weighting is 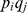 [15]. The overall 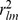 value then becomes
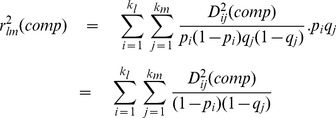
(7)


Since 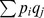 = 1, this value does not need to be normalised. And since the marginal frequencies are the same for the regular and composite tables, the same weighting applies to both.

It is interesting to contrast this weighting proportional to gene frequencies to the normal 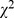 weighting of allele pairs for a 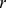
×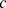 table. The 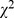 with 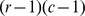 degrees of freedom can be expressed as the sum of 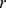
×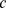 individual 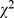 values each with 1 df, if the values are weighted by 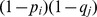 rather than 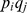. Thus the 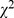 weighting gives rare alleles higher weight than common ones. Zhao et al [15] have compared these two measures, amongst others, for their use in QTL mapping, and recommend a standardised 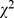 weighting for this case. However the higher weighting for rare alleles, as suggested from 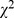, performs poorly as just a simple measure of LD (Appendix S2).

Because of the different weighting for 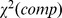 and 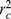, there is no simple relationship between the two statistics. In general, however, significant values of 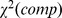 will lead to low estimates of 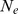 and non-significant values of 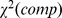 will be associated with high 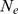 estimates. See [16] for a more detailed examination of the 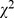 statistic.

#### The estimation of 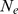


The theory for estimating 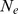 from unlinked loci has been developed by Weir [8], Weir and Hill [6] and Hill [3]. The effective size refers to a model Wright-Fisher population, and departures from this model, such as permanent pair bonding, make a difference of a factor of 2 in 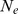 estimates [6]. Such pair bonding is, of course, unlikely in fruit fly populations. A model assuming discrete generations as considered here is, however, necessarily an approximation to real populations that are likely to have overlapping generations.

Taking no account, for the moment, of the effect of sample size, the key equation relating the expected LD level to 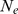 is
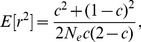
(8)where 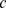 is the recombination frequency. This reduces to
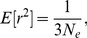
(9)for unlinked loci, 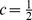. The expectation for 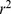 here assumes a balance between increase of 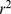 due to finite population size and loss due to recombination. All of the equations below assume this balance between drift and recombination. Equation (8) is derived using the ratio of expectations of 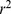 rather than the expectation of the ratio (see Hill [17]). However computer simulation shows that it works well for loosely linked or unlinked genes, those of interest in the present study. It is unbounded for low values of 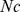, when the expression given by Sved and Feldman [18]:
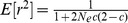
(10)seems to work better. However for 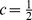, the RHS of equation (10) reduces to 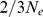, which is double the value of equation (9) and clearly inaccurate at this end of the scale.

Equations (8)–(10) assume the measurement of haplotype or gamete frequencies. As previously indicated, diploid data may be taken into account using the composite LD measure. It follows from equations (1) and (4) that the expectation for this measure is identical to that of (8):
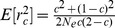



Sample size is a critical issue in determining LD levels [8], [6], [3]. This is especially the case for unlinked loci, where the levels of 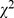 and 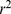 cannot be zero even if there is no association of loci in the population being sampled. The usual procedure in estimating true LD levels in the population is simply to subtract the level of 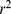 expected for zero LD with a particular sample size. As pointed out in [19], however, there is one circumstance where this procedure will not work. With complete LD in the population, 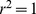, as commonly found for the most tightly linked SNPs, the subtraction will falsely suggest 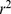 levels less than 1.

The effect on the equation for gametes (8) is to increase the expected value of 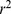 by a factor of 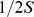, where 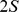 is the haploid sample size. The 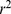 statistic in this case is shown as 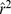 to indicate that it is an estimate that includes the effects of sampling
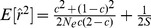
(11)


In fact the exact expectation for 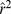 should include the term 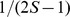 rather than 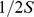, equivalent to noting that the exact expectation of 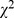 is 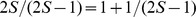 rather than 1 [20]. Weir [8] takes this factor into account in working with the ‘unbiased’ rather than ‘biased’ value of 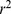.

As shown in equations (1) and (3) of Appendix S1, the expectation for the composite 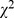, or equivalently the composite LD coefficient 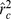, involves the factor 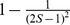, rather than 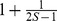 applicable to haploid data. This factor is very close to 1. Similarly the sampling correction factor for 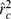 for a diploid sample of size 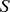 is close to 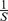:
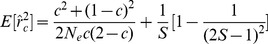
(12)or for unlinked loci:



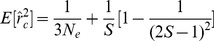
(13)The estimate for 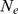 comes from inverting equation (13), where 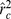 is calculated according to equation (6) and each 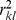 is calculated according to equation (7)
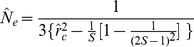
(14)


#### The effect of null alleles

Use of the composite disequilibrium index depends critically on the ability to distinguish heterozygous and homozygous genotypes. Unfortunately the presence of any null alleles makes this distinction difficult. Genotypes such as 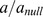, will be incorrectly scored as 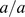. Homozygous null genotypes are not easily detected, since it is difficult to distinguish between absence of a band and simple failure of the PCR reaction in the rare cases expected for homozygotes.

The expected effect of null alleles on the composite LD statistic can be quantified as in Appendix S3. This shows that a null allele at one of the two loci at a frequency 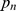 alters the expectation of equation (1) to:
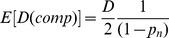



The statistic 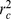 is increased by the factor 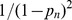.

Although this effect may be small, it can readily be shown to overwhelm the calculations when the expected LD value is small due to high effective population size. In the case of an infinitely large population, the true value of 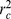 is expected to be just the sampling correction, which is approximately 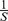. A null allele at one of the two loci is expected to increase this value to 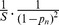. Applying equation (13), the estimated value of 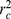 is then found by subtracting the usual 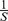 sampling contribution, giving
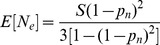
(15)


Applying numerical values to equation (15), for a sample size 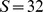 and null frequency 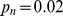, the equation yields a value for 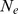 of 259. The actual population in this case should be infinitely large, so that a null allele frequency as low as 2% can have a strikingly large effect. A null allele at frequency 0.1, still difficult to detect, leads to a 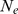 estimate of 45.

#### Simulations with null alleles

Simulations with null alleles have been carried out to test these expectations. These are 2-locus simulations with heterozygosities ranging from 50% to 87%. Under these conditions, equation (15) may slightly over-estimate the effect of null alleles. For example, in the above case with 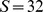 and 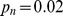, simulation yields a value of 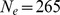 compared to expectation of 259, while 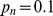 yields 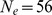 compared to 45.

Simulation can also be used to check on more realistic cases where the value of 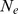 comes from multiple loci, rather than a two-locus simulation. These show that even low levels of null alleles at a single locus may have measurable effects. For example with 32 loci each with 5 alleles, the presence of just one locus amongst these having a null allele frequency of 10% can have a detectable effect, reducing the expected value of 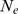 from infinitely large to less than 1,000. Much the same result is found for 5 loci each with a null frequency of 2%. Simulations also indicate that 8 out of 16 loci having null alleles at a particular frequency has much the same effect as one out of two loci in the simulations and calculations given above.

#### Correcting the effect of null alleles through permutation

A general formulation for the estimation of 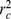 may be given as follows:
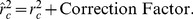
(16)


Here 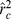 is the estimate derived from the data, and 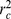 is the true measure of LD in the population, which is the quantity of interest in estimating 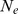. The analysis above has shown that in the absence of null alleles, the correction factor is attributable purely to sampling, and is 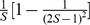. The analysis on null alleles has shown that these will act as disturbing factors, whose effect can conveniently be subsumed into the correction factor in equation (16).

A randomising procedure can be suggested that will ameliorate the effect of null alleles. If the genotypes at each locus are independently randomly permuted amongst individuals, such as in the exact test of significance of LD, eg. [21], there can be no underlying LD. So the mean value of 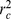 given by the average of many such randomly permuted samples is a direct estimate of the correction factor in (16) taking into account the actual genotype structure. If 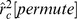 is the estimated value of 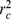 in such permuted samples, then equation (16) becomes
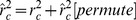
(17)


From equation (9), the estimate of 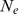 is then simply
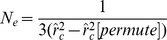
(18)


Both 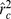 and 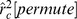 can be given with or without the sampling correction factor 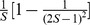. In the data tables below, the factor has been subtracted from both in order to use equation (14) to estimate the value of 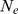 with no permutation. However for the value of 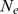 with 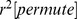 subtracted, the sampling factor cancels out and could have been omitted.

The permutation approach can be tested by simulation. This is shown in the first four lines of Table 2. All, except for the final two rows, involved 16 loci simulated for 20 generations, followed by sampling of 32 individuals. The first row shows the average 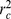 value for a range of population sizes from 32 to 1028. The second row shows the estimated 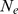 values using equation (14), with each of the 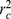 values calculated directly from the composite haplotype table according to equations (1) and (4). The 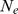 values are in good agreement with expectation.

**Table 2:** Observed statistics from simulations with and without incorporating single-locus disequilibrium.

Actual *N_e_*	32	64	128	256	512	1024
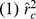	0.00993	0.00511	0.00255	0.00129	0.00065	0.00032
(2) *N_e_* (from equation 14)	34	65	131	259	516	1036
(3) *N_e_* (null alleles)	26	41	59	76	89	97
(4) *N_e_* (null alleles+permute)	33	64	127	249	494	1025
(5) 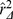 (diseq. included)	0.01067	0.00598	0.00352	0.00225	0.00163	0.00133
(6) *N_e_* (diseq. included)	31	56	95	148	203	249
(7) *N_e_* (diseq. included+permute)	35	68	134	265	523	1040
(8) *N_e_* (null alleles - diseq. included)	31	56	96	147	206	248
(9) *N_e_* (nulls - diseq. corr.+permute)	35	68	136	274	559	1127
(10) 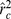 s.d.	0.00655	0.00397	0.00285	0.00231	0.00205	0.00193
(11) 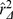 s.d. (diseq. included)	0.00468	0.00272	0.00186	0.00146	0.00126	0.00117
(12) 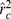 s.d. (32 loci)	0.00454	0.00277	0.00195	0.00153	0.00134	0.00124
(13) 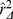 s.d. (diseq. included, 32 loci)	0.00299	0.00167	0.00108	0.00081	0.00067	0.00059

All used sample size S = 32.

The effect of introducing null alleles is shown in row (3). The simulations here involved choosing 8 of the 16 loci, and replacing 5% of alleles with null alleles in these. The 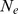 values calculated using equation (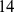) are drastically reduced, especially for the higher population sizes. However the permutation correction in row (4) essentially brings the estimated 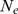 values back to their expected value.

In the case of an infinitely large population, simulation is not necessary to justify the permutation approach for correcting for null alleles. The loci would be in linkage equilibrium in such a population, with a true value of 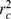 of zero. The only contributing factor to the observed value of 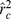 must be the correction factor, attributable to null alleles, plus the usual sampling factor of approximately 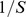. Additional permutation of genotypes in a sample from a population with zero LD will not have any effect, so the 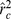 estimates with and without permutation will be identical and equal to 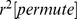.

The case of an infinitely large population also serves to show that the permutation approach will NOT work in removing biases due to non-random mating. For example, a sample might consist of individuals from two independently randomly mating populations, where the substructure has not been recognised. Such a sample will give a reduced estimate of 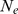 due to the induced LD [22] even though there may be no LD within each of the two contributing populations. However permuting the sample cannot resolve this issue. It can be seen that the value of 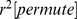 from the composite table will be zero, except for the normal sampling component of approximately 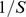, assuming no null alleles. The application of equation (17) would then falsely indicate that the LD within populations was real and attributable to small population size. A valid correction could be produced if the sub-samples from the two populations could be independently permuted, which is possible in computer simulation but not with real data where the substructure is unknown.

Taking account of all types of departure from random mating thus appears difficult. But Waples and England [23] have considered the case of migration into a random mating population, and shown that there is little effect on 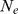 estimates in this case.

#### Including the single-locus disequilibrium factor

As mentioned above, a homozygosity correction term was suggested by Weir [8], as shown in equation (5). The effects of this term are shown in row (5) of Table 2, the 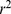 value, and row (6), the 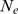 value. The latter shows a substantial bias in 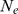 values, especially for the larger population sizes. The size of this discrepancy seems surprising, since, under random mating, the mean value of the homozygosity correction should be zero, and only a small correction should result. However there is a bias due to the fact that, in a finite-size sample, the expectation of 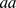 frequency is less than 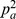. This is most evident where there is a single 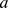 allele, giving 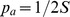, but where the frequency of the 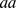 genotype must be zero.

The obvious way of eliminating this bias would seem to be the use of 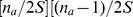] as the expected frequency of homozygotes. But simulation shows that this substantially over-corrects the bias. It is, however, possible, just as in the case of correcting the bias for null alleles, to use a permutation correction. This involves calculation of 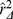 from equation (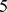), random permutation of genotypes in the sample, and calculation of 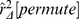 in permuted samples. The procedure may be summarised as:
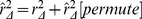
(19)


From equation (19), the estimate of 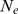 is
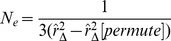
(20)


Simulation in row (7) of Table 2 shows that this correction works well for all 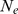 values.

The homozygosity deviation factor, 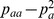, was not specifically designed in [8] to take into account null alleles. It seems particularly vulnerable to their effect, since 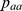 may be substantially over-estimated. However simulation shows that this factor dramatically improves rather than worsens the effect of null alleles. In contrast to the bias of the 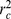 considered previously that lacks the disequilibrium correction, row (8), which introduces null alleles at the same frequency of 5% in half of the loci, gives almost the same 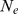 value as row (6) where there are no null alleles. As previously, the bias due to the factor can be eliminated by subtracting the permutation 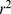 using equation (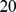), as shown in row (9).

A second advantage of the disequilibrium factor is that it reduces the variance of estimates. The 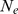 estimates given in Table 2 are based on large numbers of replicates. However the variability between individual simulation runs is high. Estimated standard deviations of 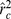 and 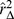 are given in rows (10) and (11). Both standard deviations are high in relation to the mean, but that associated with 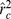 is especially so. Of course the magnitude of the standard deviations is heavily dependent on the choice of number of loci and heterozygosity levels. Doubling the number of loci from 16 to 32 substantially reduces standard deviations, row (12) and row (13), but the relativities between the two terms are maintained.

In summary of Table 2, only the original 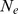 estimate from equation (14), where 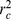 lacks the single-locus disequilibrium factor, gives unbiased 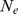 estimates. Nevertheless there is a strong reason for including the faxtor, provided that the bias in 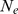 values is compensated, either by permutation as above, or by empirical correction as implemented in the computer program LDNe [9]. Weir’s insight in introducing this factor is vindicated by the increased accuracy of estimation and lowered sensitivity to null alleles.

## Results and Discussion

Results for the East coast populations are given in Table 3. Populations with low sample numbers, 15 or less, were omitted from the analysis, leaving 40 out of the original 52 samples. The table includes mostly samples from outbreak areas where the flies were not normally found, but also ten samples where the flies are endemic, including one from Queensland, the home range of the flies. The expectation is that these ten are samples from large populations.

**Table 3:** Summary of *N_e_* estimated by various procedures for East coast outbreak populations of *B.tryoni*, with the most likely estimate shown by ⇓.

	S	No homozygote correction		Homozygote correction			Likelihood Significance	
		Unlinked No permute Eqn. (14)	Unlinked permute Eqn. (18)	Unlinked permute Eqn. (20) ⇓	All loci			
					permute Eqn. (20)	LDNe	genotype	composite
Albury03	27	60	∞	∞	∞	∞		*
Barooga03	33	40	30	40	20	20		***
Condobolin02	42	40	∞	∞	∞	∞	*	
Coota02	43	110	∞	450	340	510		***
Corowa02	22	20	120	180	100	∞		
Cowra	20	20	230	150	180	∞		
Deniliquin02	40	30	40	40	30	∞	***	***
Deniliquin03	53	40	100	150	70	90	*	***
Deniliquin04	73	50	130	160	70	110	*	***
Dubbo02	26	30	180	130	160	∞		**
Forbes02	34	40	250	180	170	∞		**
Grenfell02	31	130	∞	∞	∞	∞		***
Hay02	26	20	30	20	20	140		***
Hay03	28	40	230	120	50	80	*	***
Henty02	20	20	120	60	50	190		**
LakeCarg02	74	30	40	50	30	70	**	***
Leeton03	82	70	110	160	70	80	**	***
Narrandera04	25	30	∞	770	130	510		*
Parkes02	20	30	130	100	80	500		
Parkes03	41	30	140	140	190	310		**
Temora02	20	20	120	160	150	∞		
TheRock02	20	30	410	170	100	∞		
Tumut	20	20	670	470	270	∞		*
Wagga02	57	70	790	∞	∞	∞		
Wagga03	162	210	660	740	610	860		
Wahgunyah	24	20	90	70	50	∞	*	*
Wilcannia02	43	20	50	60	30	50	***	***
Wodonga	42	30	110	110	100	130		***
WWyalong03	24	120	∞	∞	110	∞		*
Young02	49	110	170	380	400	440	**	***
Coffs02¶	18	40	70	60	70	∞		
Foster02¶	34	40	∞	∞	∞	∞	***	***
Grafton03¶	29	40	290	280	510	∞		***
Maclean02¶	34	50	600	280	360	∞		***
NSW03¶	42	90	380	∞	∞	∞		
QLD03¶	42	70	430	290	530	∞		***
Sawtell02¶	34	120	∞	∞	∞	∞		
SWRocks02¶	33	40	∞	∞	∞	∞		***
Syd03¶	42	130	∞	∞	∞	630		*
Taree03¶	30	40	∞	∞	∞	∞		

¶ Non-outbreak population.

* Significant at 5% level.

** Significant at 1% level.

*** Significant at 0.1% level.

The results are based on 29 microsatellites, a total of 29×28/2 = 406 locus pairs. Because of missing readings, not all pairs are present in all populations.

Amongst the 29 loci, 5 pairs are known to be closely linked, 51 pairs to be loosely linked, and 197 to be unlinked [24]. For the remaining 153 locus pairs, one or both chromosomes are unknown. Average values of 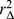 for the four classes are 0.0434, 0.0153, 0.0084 and 0.0096 respectively. As expected, average values are higher for the known linked loci.

Values of 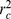 were calculated from the composite haplotype tables, and 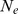 values (column 3) were then calculated from these values using equation (14). All populations, including the eight non-outbreak populations, show very low estimated population sizes. All are highly significantly different from infinite population size. The major conclusion from the above analysis, however, is that the existence of either null alleles or population sub-structure can cause cause 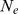 values to be substantially under-estimated.

A direct test for null alleles is given in Table 4. The signal for null alleles is, eg. [25], excess of homozygotes over expectation. In a data set with multiple populations, a non-parametric test can be carried out based on number of populations where there is such an excess. Table 4 shows the results, revealing at least 10 out of 29 microsatelltes with significant excess of homozygotes, which, in the lack of systematic homozygote excess, can likely be attributed to null alleles rather than to population structure.

**Table 4:** Excess of homozygosity for different microsatellites.

Rank	Microsatellite	Number of populations	
		Homozygous excess	Out of
1	Bt2.9a	36	39
2	Bt6.1a	33	36
3	Bt15	36	40
4	Bt4.1a	36	40
5	Bt1.7a	35	40
6	Bt2.6a	33	40
7	Bt2.6b	31	38
8	Bt3.2b	30	37
9	Bt1.6a	31	39
10	Bt32	30	39
11	Bt10	30	40
12	Bt7.9a	29	39
13	Bt6.12a	27	40
14	Bt5.10a	27	40
15	Bt8.5a	26	40
16	Bt11	25	40
17	Bt7.2b	23	39
18	Bt1.1a	20	40
19	Bt9.1a	20	40
20	Bt14	18	40
21	Bt8.6a	18	40
22	Bp78	18	40
23	Bt17	17	40
24	Bt4.3a	16	40
25	Bt4.6a	15	38
26	Bt6.8a	15	40
27	Bt8.12a	15	40
28	Bt6.10b	14	40
29	Bt5.8a	9	38

Returning to Table 3, column 4 shows the values of 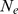 using 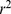 values corrected using equation (17). The correction factor in this case comes from 200,000 simulated populations for each outbreak sample. The 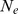 values clearly have a more realistic mixture of population sizes than the estimates based on the raw 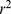 values. Positive values of greater than 1,000 are listed as infinite, as also are the 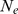 estimates associated with negative 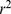 estimates. Lower values of 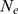 have been rounded to the nearest 10.

The disequilibrium factor is introduced in column 5. This column is marked as giving the most likely estimate of 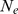. As expected, all of the really small population size estimates come in the outbreak populations rather than in the endemic populations.

The 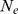 values in columns 3–5 are based on the unlinked locus pairs, including the 153 additional pairs likely to be loosely linked or unlinked. The values in column 6 are the equivalent corrected 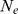 estimates based on all locus pairs. These can be directly compared to the values of 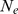 given by the LDNe program [9], also based on all locus pairs. There is good agreement for the smallest population sizes, although the LDNe program shows infinite sizes in a number of cases where the values of 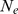 in column 5 are finite.


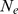
 values in column 5, using unlinked loci, differ very little from values on column 6 using all loci. The expectation is that the use of linked loci will lead to under-estimation of 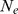. Many, but not all, values in column 6 are slightly below those in column 5, but the differences are not large. This result seems fortuitous, given that linkage relationships are not as well established for many organisms, necessitating the use of all locus pairs.

The final two columns of Table 3 show two different tests of significance, each based on the unlinked plus likely unlinked sub-sample of locus pairs. The first is the usual genotype likelihood test of LD [21], based on permutation of genotypes, with log likelihoods of the genotype tables summed over all relevant locus pairs. The second is a likelihood test based on permutation of genotypes, with likelihoods calculated on the composite haplotype tables. This test seems much more sensitive. Partly this is because, as indicated above and illustrated in Figure 2, the composite haplotype table is much denser than the genotype table, where all the zero and unit values do not contribute to the likelihood. However the second test is influenced by LD, but also by null alleles. The significant values are mostly associated with low population sizes, but there are exceptions to this in both directions. In general, the significance tests seem to be of limited value in judging whether population sizes are infinite or not.

The results from North-West samples [11] are given in Table 5. The results show a comparable proportion of high population numbers compared to the East coast populations of Table 3. Less has been known about these populations, but these results would suggest that, with the exception of the final two samples from Broome and Derby in West Australia, these are well-established outbreaks in most cases.

**Table 5:** Estimated *N_e_* values for North-West population samples.

	S	No homozygote correction		Homozygote correction			Likelihood Significance	
		Unlinked No permute Eqn. (14)	Unlinked permute Eqn. (18)	Unlinked permute Eqn. (20) ⇓	All loci			
					permute Eqn. (20)	LDNe	genotypea	composite
K-Ke2002	22	30	160	270	90	∞		***
K-Ke2003	39	20	60	90	100	∞		***
K-Kl2000	77	70	240	290	160	190		
K-Kl2001	50	60	190	210	170	∞		
K-Kl2002	44	30	60	100	70	80	**	***
K-Kl2003	50	50	∞	∞	∞	∞		**
K-Km2002	27	20	420	280	90	50	*	***
N-DWN02	40	20	50	80	90	780		
N-DWN03	20	60	∞	∞	∞	∞		***
N-DWN99	20	∞	∞	∞	∞	∞		
N-DWNBUSH02	30	40	∞	∞	∞	∞		
N-DWN-KTH03	19	60	∞	∞	∞	∞		
N-GOVE02	17	∞	∞	∞	∞	∞		
N-KAK02	40	40	80	120	120	440		***
N-KTH03	20	30	100	230	∞	∞		
N-KTHGO02	28	80	∞	440	470	∞		**
N-mDK02	27	40	300	180	270	∞		
N-mDKA02	20	80	∞	∞	150	∞		
N-mKKu03	36	30	100	120	80	200		**
N-nDWN02	50	70	140	210	320	∞	*	
N-nDWN03	20	90	∞	∞	∞	100		***
N-nKTH03	20	30	170	270	420	∞		
Q-AT02	21	40	∞	∞	∞	∞		
Q-ATH99	21	110	∞	∞	∞	340		
Q-CT00	23	140	∞	∞	∞	∞	*	
Q-CT99	17	50	90	280	∞	∞		
Q-LR00	24	80	∞	∞	∞	110		
Q-MB02	21	40	∞	∞	∞	∞		
Q-Qld00	94	110	260	260	390	∞		***
Q-QLD01	55	70	280	280	630	300		
Q-QLD02	40	40	220	250	160	∞	*	
Q-QLD03	42	40	250	110	140	∞		
W-Brm01	21	20	30	40	30	80		
W-Der01	17	10	10	10	10	10	***	**

* Significant at 5% level.

** Significant at 1% level.

*** Significant at 0.1% level.

### Summary of the Findings

The Burrows composite index can be equivalently derived from a ‘composite haplotype table’ in which all genotypes sampled contribute four possible haplotypes.

Although the composite haplotype table has marginal totals that are even numbers due to double counting, a valid 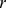
×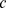 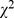 can be calculated for the table. The 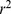 value calculated from this table, 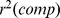, needs to be multiplied by a factor of 4 to give 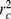, a valid estimator of 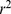.

The expected 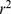 value calculated for the table is 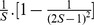 in the absence of LD. This contrasts with the sampling correction of 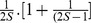 for 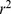 calculated when haplotypes can be recognised.

The overall calculation of 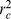 involves summation of values from different locus pairs. Within locus pairs, it involves summation of 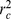 values for each pair of alleles. The weighting for the former is taken from [9], while a weighting proportional to gene frequencies is proposed for the latter.

The results when this formula are applied to data from Queensland fruit fly give low 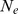 values in all samples, including ones from known large endemic populations. Null alleles are suggested as a cause for this discrepancy, and shown to be frequent in the data.

The effect of a null allele at frequency 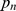 is shown to increase the composite 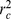 value by the fraction 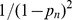. Although this effect seems small, it will nevertheless overwhelm the calculations for large population sizes.

The 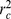 value can be corrected for null alleles using a comparison between the calculated 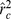 value and an equivalent 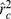 value calculated when genotypes in the sample are permuted at random. This correction is verified by simulation.

The single-locus disequilibrium factor suggested by Weir [8], equivalent to a homozygosity correction, is introduced into the calculation. This alters the value of 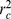 to 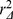. Use of 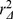 is shown to bias the 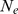 values due to the difficulty of calculating the single-locus disequilibrium factor using 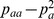 in a finite population.

Simulation shows that this bias can be rectified using the same permutation approach as for null alleles.


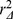
, and 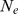 calculated from 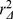, have lower variances than 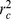, and 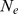 calculated from 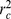.

Simulation shows that the 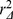 values are almost unaffected by null alleles, in sharp contrast to the 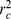 values.

The estimates of 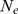 from both East coast and NorthWest populations are, as expected, mostly low for outbreak populations and high for endemic populations.

The calculations are based on loci known to be unlinked, but are not substantially changed when all locus pairs are considered. Linkage information is usually not available for non-laboratory organisms, and this result shows that lack of such information may not be critical in calculating 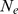 based on LD.

Although the LDNe program [9] is empirically based, it uses the single-locus disequilibrium factor, and appears to work well both with and without null alleles.

## Supporting Information

Figure S1 **The effect on the estimate of r2 from ?2 weighting compared to allele frequency weighting when introducing a single new mutant.**
(TIF)

Appendix S1 ***x*** **^2^ and** ***r*** **^2^ for the composite haplotype table.**
(PDF)

Appendix S2 ***x*** **^2^ as a measure of LD for multiple alleles.**
(PDF)

Appendix S3 **The effect of null alleles on r^2^.**
(PDF)

Data S1 **Microsatellite data for East coast samples of** ***Bactrocera tryioni*** **(Queensland Fruit Fly).**
(TXT)

Data S2 **Microsatellite data for NorthWest samples of** ***Bactrocera tryioni*** **(Queensland Fruit Fly).**
(TXT)
