# Supplementary material for: Estimating Effective Population Size from Linkage Disequilibrium between Unlinked Loci: Theory and Application to Fruit Fly Outbreak Populations
Source: PLoS One. 2013 Jul 23;8(7):e69078. doi: 10.1371/journal.pone.0069078 (PMC3720881; doi:10.1371/journal.pone.0069078)
Supplement: Appendix S1 — x 2 and r 2 for the composite haplotype table. (PDF) [file pone.0069078.s002.pdf]

Appendix S1. χ^2 and r^2 for the composite haplotype table

Setting aside for the moment the complications of double-counting, the composite haplotype table of Figure 1 can be treated as a regular 2 x 2 contingency table. A one degree-of-freedom χ^2, designated $\chi^2(comp)$, can be calculated. If the number of ab haplotypes, $4n_{11} + 2n_{12} + 2n_{21} + n_{22}$ is given the designation M, then

$$\begin{aligned}\chi^2(comp) &= \frac{4S(4S.M - 2n_a 2n_b)^2}{2n_a.(4S - 2n_a).2n_b.(4S - 2n_b)} \\ &= \frac{4S(M.S - n_a n_b)^2}{n_a.(2S - n_a).n_b.(2S - n_b)}\end{aligned}$$

Gamete frequencies cannot be calculated in terms of just the n values of Figure 1, since the n_{22} parameter contains both $ab/--$ (coupling) and $a-/-b$ (repulsion) genotypes. The frequencies of these two types, which usually cannot be observed, may be given the designation n_{22c} and n_{22r}, where $n_{22c} + n_{22r} = n_{22}$. It is convenient to introduce the parameter $\alpha = (n_{22c} - n_{22r})/2$. Then the numbers of coupling and repulsion genotypes become $n_{22}/2 + \alpha$ and $n_{22}/2 - \alpha$ respectively. The gamete numbers are as shown in Table S1.

The one degree-of-freedom $\chi^2(hap)$, testing haplotypes from Table S1 is

$$\begin{aligned}\chi^2(hap) &= \frac{2S[2S(M/2 + \alpha) - n_a n_b]^2}{n_a.(2S - n_a).n_b.(2S - n_b)} \\ &= \frac{2S(M.S - n_a n_b)^2}{n_a.(2S - n_a).n_b.(2S - n_b)} + \frac{2S(2N\alpha)^2}{n_a.(2S - n_a).n_b.(2S - n_b)} + \alpha \text{ term} \\ &= \frac{1}{2}\chi^2(comp) + \frac{2S(2S\alpha)^2}{n_a.(2S - n_a).n_b.(2S - n_b)} + \alpha \text{ term},\end{aligned}$$

from which

$$\chi^2(comp) = 2\chi^2(hap) - \frac{4S(2S\alpha)^2}{n_a.(2S - n_a).n_b.(2S - n_b)} - \alpha \text{ term}$$

These terms allow calculation of the expectation of $\chi^2(comp)$, E[$\chi^2(comp)$], over repeated sampling of S diploid individuals. The term in α has zero expectation. The regular 2 x 2 one degree-of-freedom $\chi^2(hap)$ has expectation 2S/(2S - 1) [20].

The term in α^2 is more difficult. The numerator of this term can be shown to have the expectation $(2S)^4 p_a(1 - p_a)p_b(1 - p_b)$, where p_a and p_b are gene frequencies at the a and b loci. The denominator similarly can be shown to have the expectation $(2S)^2(2S - 1)^2 p_a(1 - p_a)p_b(1 - p_b)$. Although there is a positive covariance between the numerator and denominator of this α^2 term, computer simulation has shown that the expectation of the ratio is extremely close to the ratio of expectations, which is $(2S)^2/(2S - 1)^2$.

Overall, therefore,

$$\begin{aligned}E[\chi^2(comp)] &= 2.\frac{2S}{2S - 1} - \frac{(2S)^2}{(2S - 1)^2} \\ &= 1 - \frac{1}{(2S - 1)^2} \qquad (1)\end{aligned}$$

The $\chi^2(comp)$ statistic thus has expectation very close to unity, and is less biased than the haploid χ^2 [20], [8]. Computer simulation also shows that the distribution of the $\chi^2(comp)$ statistic is close to expectation in significance tests at the 5%, 1% and 0.1% levels. Results from 10^8 replicate samples of size S = 32 set up with zero LD are shown in Table S2. In each case the observed significance levels are closer to expectation for the composite χ^2 than for the χ^2 calculated from just the known gametes.

The above calculations have all been for 2 x 2 tables. They extend to r x c using the regular weighting for χ^2, e.g. [17]

$$\chi^2_{(r-1)(c-1)} = \sum_i \sum_j \frac{D_{ij}^2}{p_i(1-p_i)q_j(1-q_j)}.(1-p_i)(1-q_j) \quad (2)$$

Because the marginal totals for the composite table are just a multiple of 2 of the regular table, the same summation argument applies to both.

A similar expectation applies to r_c^2 as to $\chi^2(comp)$. For a 2 x 2 table, with observed total S:

$$\chi^2 = S.r^2$$

Because the weighting of contributions to $\chi^2(comp)$ and r_c^2 are different, cf. equation (2) above versus equation (7) of the main text, there is no simple relationship between the two. However the expectations for the components of r_c^2 and $\chi^2(comp)$ differ only by the factor S, and the overall expectation for r_c^2 is given from (1) as

$$E[r_c^2] = \frac{1}{S}.[1 - \frac{1}{(2S-1)^2}] \quad (3)$$

The validity of equations (2) and (3) can be tested by simulation. For the $\chi^2(comp)$ calculation, a set of approximately 10^{10} simulations with $S = 32$ gave a mean value of 0.999741 $\pm$ 0.000070 compared to expectation of $1 - 1/63^2 = 0.999748$. As pointed out in Table S2, the distribution is also very close to a χ^2 distribution.

Table S1 Numbers of the four haplotypes in terms of n values shown in Figure 1 and the parameter α giving the (unobservable) difference between coupling and repulsion genotypes.

	b	-	Total
a	$2n_{11}+n_{12}+n_{21}+\frac{1}{2}n_{22}+\alpha$ $=M/2+\alpha$	$2n_{13}+n_{12}+n_{23}+\frac{1}{2}n_{22}-\alpha$	n_a
$-$	$2n_{31}+n_{21}+n_{32}+\frac{1}{2}n_{22}-\alpha$	$2n_{33}+n_{23}+n_{32}+\frac{1}{2}n_{22}+\alpha$	$2S-n_a$
Total	n_b	$2S-n_b$	$2S$

Table S2 Observed and expected numbers of significant deviations from expectation from χ^2 values calculated from replicate sampling with S = 32 from an infinitely large population in linkage equilibrium.

		Significance level	
	5%	1%	0.1%
Observed (composite)	5,023,330	998,632	98,622
Observed (haploid)	5,061,141	958,095	80,383
Expected	5,000,000	1,000,000	100,000
