# Supplementary material for: Estimating Effective Population Size from Linkage Disequilibrium between Unlinked Loci: Theory and Application to Fruit Fly Outbreak Populations
Source: PLoS One. 2013 Jul 23;8(7):e69078. doi: 10.1371/journal.pone.0069078 (PMC3720881; doi:10.1371/journal.pone.0069078)
Supplement: Appendix S2 — x 2 as a measure of LD for multiple alleles. (PDF) [file pone.0069078.s003.pdf]

Appendix S2. χ^2 as a measure of LD for multiple alleles

An undesirable consequence of the χ^2 weighting of r^2 is shown in Figure S1. This considers a 2-allele model with complete association between genes at the two loci ($r^2 = 1$, $\chi^2 = 2N = 100$). A single mutation to a new allele occurs at the a locus. The average value of r^2 estimated using χ^2 weighting falls from 1.0 to 0.5. On the other hand the average value of r^2 estimated using equation (22) is reduced only slightly, to 0.97. The χ^2 weighting of individual r^2 values gives inappropriate weighting to low frequency alleles compared either to a straight averaging or using the frequency-weighted averaging recommended here.
