# Supplementary material for: Estimating Effective Population Size from Linkage Disequilibrium between Unlinked Loci: Theory and Application to Fruit Fly Outbreak Populations
Source: PLoS One. 2013 Jul 23;8(7):e69078. doi: 10.1371/journal.pone.0069078 (PMC3720881; doi:10.1371/journal.pone.0069078)
Supplement: Appendix S3 — The effect of null alleles on r2. (PDF) [file pone.0069078.s004.pdf]

Appendix S3. The effect of null alleles on r^2

We consider genotype and composite gamete frequencies for the case where there is a null allele at the a locus. Six gamete genotypes are now possible, with frequencies:

Gamete:	ab	$a\_$	$\_b$	$\_\_$	nb	$n\_$
Frequency:	P_1	P_2	P_3	P_4	P_5	P_6

Frequencies of the genes at the a locus, a, $\_$ and n are respectively p_a, $p_\_$ and p_n, also summing to 1.

Table S3 gives the expected composite frequencies under random mating of all genotypes that contribute to the composite ab haplotype, using the rules outlined in connection with Figure 1. Summing the final column of Table S3, the total ab haplotype frequency, $p_{ab}(comp)$, simplifies to $P_1 - D_n/2 + p_a P_5$, where $D_n = P_1 P_4 - P_2 P_3$. Frequencies of the other three gametes can also be written down in the same terms: $p_{a\_}(comp) = P_2 + D_n/2 + p_a P_6$, $p_{\_b}(comp) = P_3 + D_n/2 + p_\_ P_5$, $p_{\_\_}(comp) = P_4 - D_n/2 + p_\_ P_6$. The sum of these four is $1 - p_n^2$, consistent with the fact that homozygous null alleles at the a locus cannot be scored.

Calculating the observed LD parameter, $D(comp) = p_{ab}(comp)p_{\_\_}(comp) - p_{a\_}(comp)p_{\_b}(comp)$, divided by the factor $(1 - p_n^2)^2$. This gives:

$$D(comp) = \frac{1}{2}\frac{D_n}{(1 - p_n)^2}$$

Assuming further that the null allele at the a locus is not in LD with alleles at the b locus,

$$D_n = D(1 - p_n)$$

from which

$$D(comp) = \frac{1}{2}\frac{D}{(1 - p_n)}$$

The calculations are considerably more complicated when there are null alleles at both loci rather than one of the two. However simulation shows that the effects on N_e are approximately what would be expected from summing the effects at the individual loci.

Table S3 Expected frequencies of *ab* composite gametes with null allele at *a* locus

Genotype	Genotype frequency	Gamete fraction	Gamete frequency
ab/ab	P_1^2	1	P_1^2
$ab/a\_$	$2P_1P_2$	$\frac{1}{2}$	P_1P_2
$ab/\_b$	$2P_1P_3$	$\frac{1}{2}$	P_1P_3
$ab/\_\_$	$2P_1P_4$	$\frac{1}{4}$	$\frac{1}{2}P_1P_4$
$a\_/\_b$	$2P_2P_3$	$\frac{1}{4}$	$\frac{1}{2}P_2P_3$
ab/nb	$2P_1P_5$	1	$2P_1P_5$
$ab/n\_$	$2P_1P_6$	$\frac{1}{2}$	P_1P_6
$a\_/nb$	$2P_2P_5$	$\frac{1}{2}$	P_2P_5
